# Liver abscesses secondary to a non-O1/non-O139 *Vibrio cholerae* bacteremia acquired in a non-coastal area: a case report

**DOI:** 10.1186/s12941-024-00764-6

**Published:** 2024-12-20

**Authors:** Coen Veenstra, Marion Kolader, Sébastien Matamoros, Kim Sigaloff

**Affiliations:** 1 Cancer Center Amsterdam, Amsterdam UMC, Vrije Universiteit Amsterdam, Amsterdam, The Netherlands; 2 Department of Medical Microbiology and Infection Prevention, Amsterdam UMC, University of Amsterdam, Amsterdam, The Netherlands; 3 Department of Internal Medicine, Amsterdam UMC Location Vrije Universiteit Amsterdam, Amsterdam, The Netherlands; 4 Amsterdam Institute for Infection and Immunity, Amsterdam, The Netherlands

**Keywords:** Case report, *Vibrio cholera*, Liver abscess, Bacteremia

## Abstract

Non-O1/non-O139 *Vibrio cholerae* (NOVC) strains are a distinct group of *Vibrio cholerae* that do not cause epidemic cholera. NOVC infections usually cause mild forms of gastroenteritis, and rarely severe (extra)intestinal infections, mostly affecting immunocompromised patients. Here, we describe the clinical course of a patient with NOVC bacteremia causing multiple liver abscesses, after drinking from a freshwater well in a non-coastal area. This case highlights the potential of a *V.* *cholerae* strain, that is phylogenetically distinct from the current pandemic cholera strain, to cause severe extra-intestinal infections, including liver abscesses.

## Introduction

*Vibrio cholerae* is well known as the pathogen responsible for cholera outbreaks throughout history. *V.* *cholerae* is a highly diverse species consisting of > 200 serogroups. The classification of the species is based upon differences in the surface O-antigen structure of the lipopolysaccharide. *V.* *cholerae* are non-halophilic gram-negative rods, common inhabitants of bays, estuaries, brackish inland lakes, and can been found in seafood, but also in freshwater. *V.* *cholerae* concentrations vary throughout the year, and have a preference for higher temperatures, thus being more prevalent in the summer months [1, 2].

Cholera-toxin producing strains of *V.* *cholerae* are capable of causing cholera, a dehydrating diarrheal disease known for its stools resembling rice water. However, of the many serogroups only O1 and O139 are known to cause outbreaks of cholera [3–5]. In 2021 the World Health Organization reported 223.370 cases of cholera, but the actual number of annual cases of cholera is estimated to be up to 4 million [6, 7].

Other strains of *V.* *cholerae* are referred to as non-O1/non-O139 *V.* *cholerae* (NOVC) and do not cause epidemic cholera. Most NOVC infections are relatively mild cases of gastroenteritis, which are usually self-limiting in nature; only a minority require hospitalization. Nevertheless, NOVC can cause severe intestinal and extraintestinal manifestations, especially in, but not limited to, immunocompromised patients, including bacteremia (rare in choleragenic vibrios) with a reported mortality rate of > 30% in NOVC bacteremia cases [8].

In this report, we present a case of a NOVC strain, causing a severe extra-intestinal infection in an 80-year male patient with a well-suppressed HIV-1 infection, after having consumed freshwater from a local well in Morocco.

This case report was prepared following the CARE guidelines and written patient consent was acquired [9].

## Case presentation

In August 2022, an 80-year-old man presented to the emergency department, directly after having returned by plane from a Berber mountain village, his birth region in Morocco. He presented with a 2-week history of watery diarrhea 6 times daily, vomiting, fever and chills. Four days prior to the complaints, he consumed fresh water from a local well. Initially, the symptoms improved after a few days, but the fever persisted with nocturnal temperature spikes, followed by worsening of diarrhea and vomiting. Due to the deterioration of symptoms and fear for dehydration, the patient decided to seek medical attention in his place of residence, Amsterdam, The Netherlands.

His medical history consisted of a HIV-1 infection with suppressed viral load (recent CD4+ T cell count: 826 (norm 404-1612) with an undetectable viral load), and gastroesophageal reflux disease for which he used a proton pump inhibitor.

On presentation, the patient was confused and physical examination revealed a temperature of 39.0 °C with chills, a pulse rate of 120 beats per minute, and a blood pressure of 110/70 mm Hg. Apart from a capillary refill time of > 2 s, mildly dry mucous membranes and profound perspiration, no abnormalities were observed. The laboratory tests (Table 1) showed signs of inflammation with an elevated C-reactive protein (218), leukocytosis (18,4) and elevated liver enzymes.

**Table 1:** Laboratory results on admission

	Value	Reference value
C-reactive protein	281 (H)	< 5
Leucocytes	18.4 (H)	4–10
Eosinophils	0.00	< 0.50
Basophiles	0.04	0.00–0.20
Neutrophils	16.44 (H)	1.5–7.5
Sodium	126 (L)	136–145
Potassium	3.6	3.4–4.9
Creatinine	119	62–134
eGFR	49 (L)	> 60
Glucose	27.8 (H)	3.9–10
Bilirubin	39 (H)	4–24
Alkaline phosphatase	295 (H)	< 134
Gamma glutamyl transferase	454 (H)	< 110
Aspartate transaminase	94 (H)	14–43
Alanine transferase	91 (H)	< 45
pH (venous)	7.42	7.35–7.45

*H* Higher than reference value, *L* lower than reference value

The urine analysis and a chest X-ray revealed no abnormalities.

The preliminary diagnosis was sepsis of unknown origin, with a gastro-intestinal focus deemed probable, given the history of diarrhea and vomiting. Specifically, the differential diagnosis included cholangitis and cholecystitis (due to the elevate liver enzymes), other common foci of infections, such as lung and urinary tract, were deemed less probable. After samples for microbiological diagnostics (blood cultures, urine, stool) were collected, treatment with intravenous (IV) fluids, antibiotics (ceftriaxone 2 g iv qd) and antidiabetics was started.

**Fig. 1:**
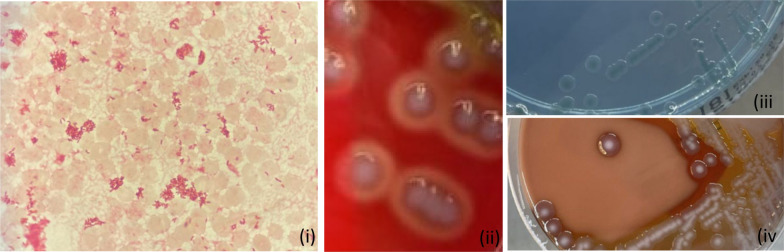
Gram stain of *V.cholerae* from positive blood culture (i); colonies of *V.cholerae* on blood agar plate, showing hemolytic colonies (ii), on Cysteine-Lactose-Electolyte-Deficient agar plate (iii), and on PolyViteX agar (iv)

Twenty-four hours after admission, blood cultures were positive for gram negative rods in 3 out of 4 vials. Following standard laboratory procedures, colonies grew on solid culture media (COS, PVX, CLED; bioMerieux, Marcy l’Etoile, France), which were initially identified as *Vibrio albensis* (score 1.85), and subsequently identified as *Vibrio cholerae* (score 2.1) by Matrix-assisted laser desorption ionization time-of-flight mass spectrometry (MALDI-TOF MS) on a MALDI-ToF Biotyper (Bruker Daltonics, Bremen, Germany) (See Fig. 1). The strain was further preliminary typed as *V.cholerae* non-O1 by agglutination with *V.* *cholerae* O1 polyvalent anti-serum (ThermoFisher Scientific, USA).

The strain was sent to the Dutch National Institute for Public Health and the Environment (RIVM) where the identification of *V.* *cholerae* was confirmed by MALDI-ToF, analyzing our institute’s sequencing data in their sequencing pipeline, and subsequent serotyping with *V.* *cholerae* O1 and O139 antiserum agglutination test, revealed a non-O1/non-O139 *V.* *cholerae* strain (NOVC). Doxycycline 100 mg IV twice a day was added to the treatment regimen as soon as the identification of the blood culture strain was made, awaiting antimicrobial susceptibility results. Using a 0.5 McFarland suspension to inoculate Mueller Hinton E agar plates (bioMerieux, Marcy l’Etoile, France) with the *V.* *cholerae* strain, a standardized set of antimicrobial agents for *Vibrio species* was tested for potential drug resistance, by using paper discs (Becton Dickinson and Company, Sparks, MD, USA). MHE-plates were incubated under ambient conditions at 35–37 °C for 18 h before reading. The *V.* *cholerae* strain was susceptible for all drugs tested, using clinical breakpoints by the European Committee on Susceptibility Testing (EUCAST Breakpoint table version 12.0, 2022), i.e. ciprofloxacin (33 mm; S ≥ 23 mm), cotrimoxazol (25 mm; S ≥ 18), cefotaxim (34 mm; S ≥ 21 mm), ceftazidim (25 mm; S ≥ 22 mm), tetracycline (26 mm; S ≥ 20 mm), and erythromycin (14 mm; S ≥ 12 mm); azithromycin susceptibility was inferred from erythromycin.

Fecal molecular diagnostic screening for bacterial causes of gastrointestinal infections (*Salmonella-, Shigella-, Yersinia-, Campylobacter species*), showed positive results for *Campylobacter spp*. After the identification of *V.* *cholerae* in de blood culture, the fecal sample was also analyzed for *V.* *cholerae* by culture using Thiosulfate Citrate Bile Salts Sucrose (TCBS) selective medium and molecular diagnostics, of which only the PCR was positive. Parasitic causes of infection, such as *Entamoeba histolytica* and *Strongyloides species* were not detected in feces and serum. Urine culture was negative.

**Fig. 2:**
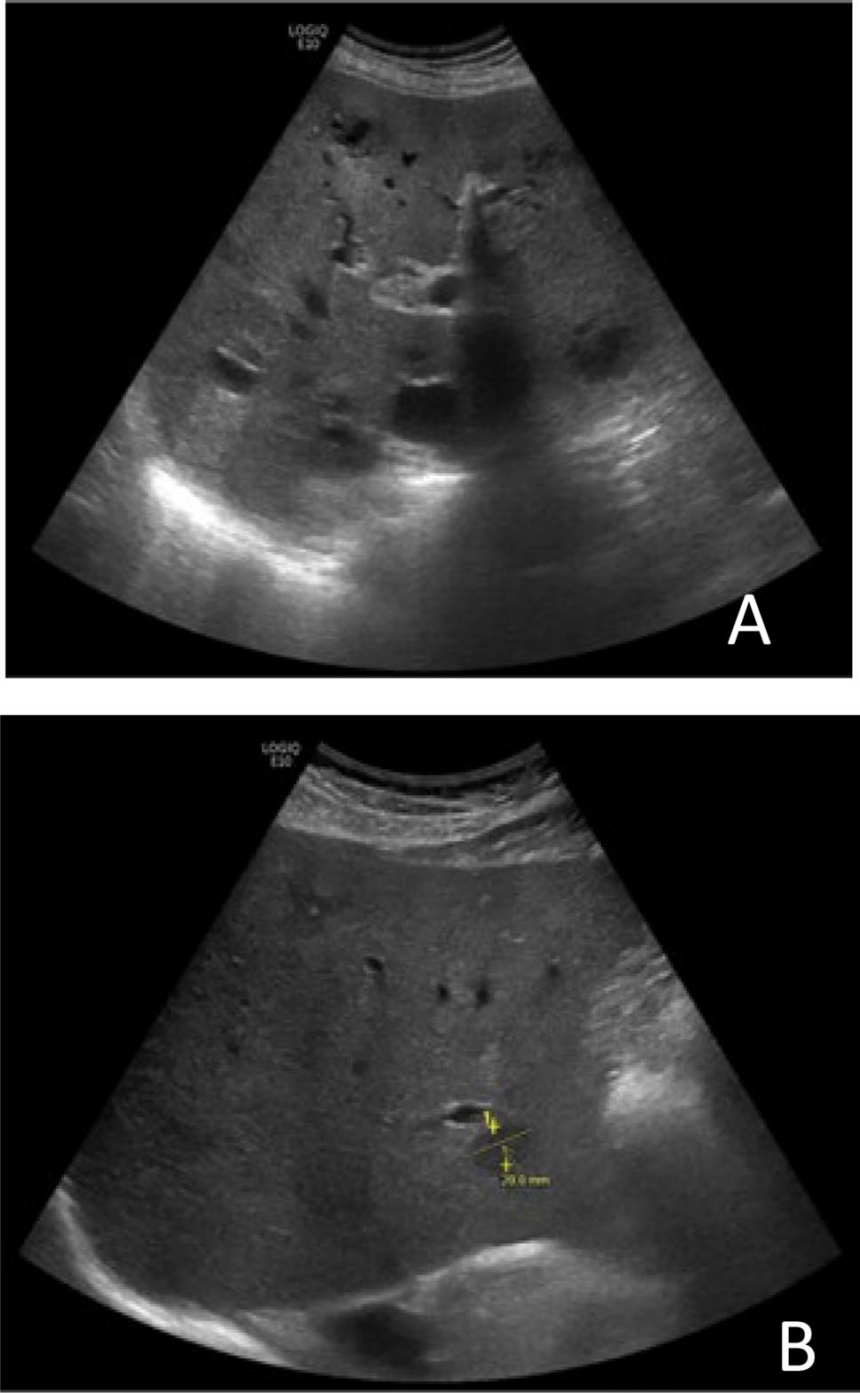
Ultrasound shows multiple liver abscesses (**A**), and residual abnormalities at the place of previous liver abscessed (**B**)

Prompted by the elevated liver enzymes, an ultrasound sonography of the abdomen was performed which showed multiple abscesses spread throughout the liver (see Fig. 2A).

Although the patient refused a diagnostic biopsy of the liver abscesses, our diagnosis was a NOVC bacteremia with multiple liver abscesses.

On day 5, the patient’s clinical situation had improved substantially, whereupon ceftriaxone and doxycycline IV were switched to oral ciprofloxacin 500 mg twice daily. On day 8 he was discharged in good clinical condition.

**Fig. 3:**
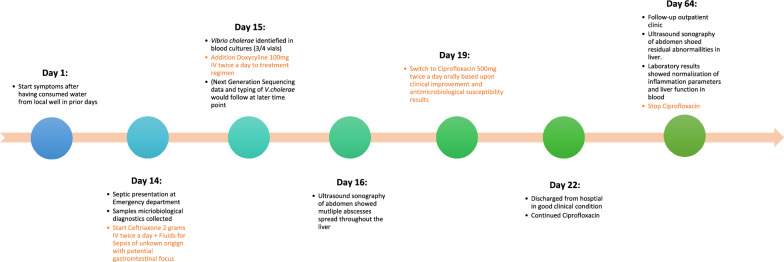
Timeline of clinical course

Six weeks after discharge, ciprofloxacin was stopped during the follow-up visit to the outpatient clinic, after ultrasound sonography of the abdomen showed only residual abnormalities at the site of previous liver abscesses (see Fig. 2B). Laboratory results showed normalization of inflammation parameters and of liver enzymes and function. See timeline for summarized clinical course (Fig. 3).

**Fig. 4:**
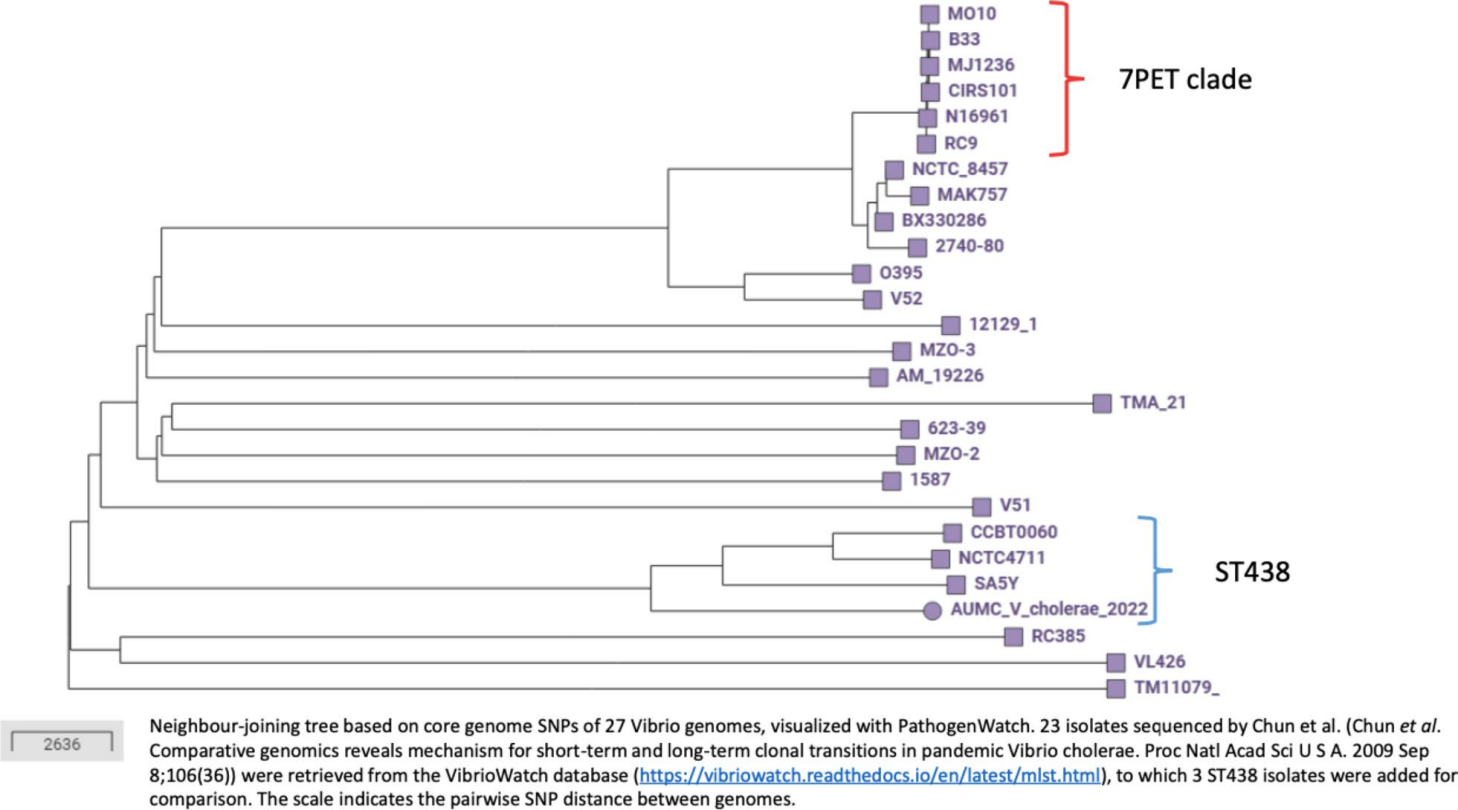
Phylogenetic comparison

Whole Genome Sequencing of the isolate revealed a high similarity to *V.cholerae* using KmerFinder [10]. The genome was subsequently uploaded to PathogenWatch (https://pathogen.watch) and analyzed using the VibrioWatch database (https://vibriowatch.readthedocs.io/en/latest/index.html). This analysis showed that the strain belonged to *V. cholerae* ST438, a rare sequence type phylogenetically distinct from the 7th pandemic cluster (see Fig. 4).

No antimicrobial resistance gene was identified. The genome contained several known pathogenicity-related genes but not the genes encoding for the cholera toxin (CT) or the colonization factor toxin-coregulated pilus (TCP), two of the major virulence factors of highly pathogenic serotypes.

## Discussion

The presented case demonstrates the potential of an NOVC strain, lacking the genes generally associated with the typical clinical presentation of cholera, to cause a severe extra-intestinal infection with liver abscesses, requiring hospitalization.

The combination of NOVC bacteremia with liver abscesses has been described before with Deshayes et al. reporting 5 cases in their review. This included cases in which NOVC was cultured from pus obtained through diagnostic biopsy of liver abscesses, proving the potential of NOVC to form liver abscesses [8, 11, 12].

Predisposing factors for severe manifestations of NOVC infections include immunodeficiency and chronic liver disease (e.g. liver cirrhosis), diabetes, HIV, malignancy, reduction of stomach acidity through medication, and advanced age [8, 13–16]. The pathogenesis of NOVC infections is less well understood, and the number of annual cases is unclear, as NOVC infections are not notifiable in many countries. However, The US Centers for Disease Control and Prevention (CDC) report approximately 100 cases of NOVC infections annually, through their Cholera and Other Vibrio Illness Surveillance initiative [17]. Furthermore, Engel et al. identified a total of 175 cases of NOVC bacteremia cases between 1980–2014, of which 25 in Europe (3 of which from The Netherlands) in their review article (2016), underscoring the rarity of the presented case [13].

At the moment, the reported incidence of NOVC infections is low, but the impact of climate change on the environment may amplify the public health risk in the future by creating more favorable conditions for the growth, spread, and persistence of NOVC bacteria (and other vibrios) in recreational waters, as observed in Finland and Sweden during the 2014 summer heat wave [2, 18, 19]. Similarly, in September 2023 the CDC issued a Health Alert Network—Health advisory notifying health care professionals about the recent reports of severe *Vibrio vulnificus* infections associated with warming coastal waters [20]. However, further research is needed to evaluate this possible correlation.

NOVC infection usually does not require therapy. In case of severe gastro-enteritis volume repletion is vital and, when deemed necessary, combined with antimicrobial therapy. As there are no controlled trials of therapy, standardized treatment for NOVC infections is currently not available. Furthermore, a high degree of heterogeneity in antimicrobial therapy strategies for systemic NOVC infections is present in the published literature, including treatment with a cephalosporin, tetracycline, or a fluoroquinolone, and combination therapy, e.g. a third-generation cephalosporin and a tetracycline for treatment of sepsis, in one study [8, 13, 15].

In our case, once the NOVC was identified in the blood cultures, the decision was made to treat with dual therapy, based upon *Vibrio vulnificus* treatment recommendations [21, 22]. Negative fecal cultures possibly reflected the fact that the fecal sample was collected after the start of antibiotics. Antimicrobial therapy was later switched to ciprofloxacin orally based upon the antimicrobial susceptibility profile, and the patient’s clinical improvement [23].

An unusual aspect of this case was the most likely route of infection, namely ingestion of contaminated fresh water. The patient was located in a non-coastal area where cholera is non-endemic, had not been eating seafood, but described the start of his symptoms after consuming water from a freshwater well. This makes it probable that the NOVC infection was acquired from this freshwater source, as opposed to the more commonly described sources [1]. It is presumed that the water source has been contaminated by someone, who travelled to a cholera endemic country or coastal area and carried the bacteria asymptomatically, or with disease. Of note, this route of infection cannot be confirmed, as testing the water from this well was not feasible.

Another extraordinary feature of this case was the presentation of NOVC with severe systemic infection and liver abscesses. Although no cultures of the abscesses were acquired, we hypothesized that these were secondary to the NOVC bacteremia. The concomitant campylobacter infection, as diagnosed by positive PCR result in feces, is an unlikely explanation for the liver abscesses, only being described in four cases worldwide (*C. curvus* 2x, *C. upsaliensis* 1x, *C. jejuni* 1x) [24–27].

In conclusion, *V.* *cholerae* is a diverse species of bacteria, best known as the cause of cholera. Infections caused by non-toxin producing, or non-O1/non-O139 strains of *V.cholerae* (NOVC) are usually asymptomatic or self-limiting, but can lead to severe illness in both immunodeficient and immunocompetent individuals. These severe systemic infections have a high mortality rate and could potentially become more prevalent in the future, due to change of *Vibrio species* concentrations in water sources.

## Key learning points

- *V.* *cholerae* non-O1/non-O139 can cause severe infections, especially in, but not limited to, patients with immunodeficiency and liver disease.
- *V.* *cholerae* non-O1/non-O139 infections might become more common in the future due to the effects of climate change
- Antibacterial therapy decisions should be made on an individual patient basis, until standardized treatment is established for NOVC infections.

## Data Availability

No datasets were generated or analysed during the current study.

## Abbreviations

NOVC: Non-O1/non-O139 *Vibrio cholerae*
HIV: Human Immunodeficiency Virus
RIVM: Dutch national Institute for Public health and the Environment
CDC: Centers for Disease Control and Prevention
